# The Adenylate Cyclase-Encoding Gene *crac* Is Involved in *Clonostachys rosea* Mycoparasitism

**DOI:** 10.3390/jof9080861

**Published:** 2023-08-18

**Authors:** Shu-Fan Yu, Zhan-Bin Sun, Shi-Dong Li, Ya-Feng Hu, Qing Ren, Jia-Liang Xu, Han-Jian Song, Man-Hong Sun

**Affiliations:** 1 School of Light Industry, Beijing Technology and Business University, Beijing 100048, China; 2 Institute of Plant Protection, Chinese Academy of Agricultural Sciences, Beijing 100193, China

**Keywords:** *Clonostachys rosea*, adenylate cyclase, gene knockout and complementation, differentially expressed genes

## Abstract

*Clonostachys rosea* is an excellent biocontrol fungus against numerous fungal plant pathogens. The cAMP signaling pathway is a crucial signal transduction pathway in fungi. To date, the role of the cAMP signaling pathway in *C. rosea* mycoparasitism remains unknown. An adenylate cyclase-encoding gene, *crac* (an important component of the cAMP signaling pathway), was previously screened from *C. rosea* 67-1, and its expression level was dramatically upregulated during the *C. rosea* mycoparasitization of the sclerotia of *Sclerotinia sclerotiorum*. In this study, the function of *crac* in *C. rosea* mycoparasitism was explored through gene knockout and complementation. The obtained results show that the deletion of *crac* influenced the growth rate and colony morphology of *C. rosea*, as well as the tolerance to NaCl and H_2_O_2_ stress. The mycoparasitic effects on the sclerotia of *S. sclerotiorum* and the biocontrol capacity on soybean Sclerotinia stem rot in ∆*crac-6* and ∆*crac-13* were both attenuated compared with that of the wild-type strain and complementation transformants. To understand the regulatory mechanism of *crac* during *C. rosea* mycoparasitism, transcriptomic analysis was conducted between the wild-type strain and knockout mutant. A number of biocontrol-related genes, including genes encoding cell wall-degrading enzymes and transporters, were significantly differentially expressed during *C. rosea* mycoparasitism, suggesting that *crac* may be involved in *C. rosea* mycoparasitism by regulating the expression of these DEGs. These findings provide insight for further exploring the molecular mechanism of *C. rosea* mycoparasitism.

## 1. Introduction

*Clonostachys rosea* is an important mycoparasite that can control numerous fungal plant pathogens according to different actions, such as mycoparasitism, antagonism, competition, secretion of cell wall-degrading enzymes, and production of secondary metabolites, including toxins and antibiotics [1]. Currently, the main research interests of *C. rosea* in the biocontrol field are focused on two aspects: the first of which is screening for more *C. rosea* strains for enhanced biocontrol capabilities under different environments. Another is investigating the molecular mechanism for *C. rosea* biocontrol, which would be beneficial for further improving the biocontrol ability of *C. rosea*. Currently, several genes, including MAPK (mitogen-activated protein kinase), transcription factor, heat shock protein 70, cell wall biogenesis protein phosphatase, nonribosomal peptide synthetase, polyketide synthase, transaldolase, and ABC transporter, have been reported to be involved in *C. rosea* biocontrol [2,3,4,5,6,7,8,9]. Among these reported genes, signal transduction genes (such as MAPK) have drawn wide attention because the signal transduction process is the preliminary step of biocontrol and can regulate other biocontrol actions. In addition to the MAPK signaling pathway, the cAMP signaling pathway is also very important and has not been studied in *C. rosea*.

The cAMP signaling pathway is conserved in fungi and can regulate their growth, development, and virulence [10,11,12]. The cAMP signaling pathway consists of several components: G-protein coupled receptors, heterotrimeric G-proteins, adenylate cyclase, cAMP-dependent protein kinase, and downstream effectors [13,14]. Adenylate cyclase is a crucial component for the synthesis of cAMP (cyclic adenosine 3′5′monophosphate) [15].

Adenylate cyclase plays an important role in biocontrol. Disruption of the adenylate cyclase-encoding gene *tac1* influences the morphology, growth, sporulation, and secondary metabolite production in *Trichoderma virens*, and the antagonism ability of mutants to pathogens of *Sclerotium rolfsii*, *Pythium* sp., and *Rhizoctonia solani* were affected [16]. The adenylate cyclase-encoding genes *BcAC* and *MrAC* from *Beauveria bassiana* and *Metarhizium robertsii*, respectively, were reported to be involved in the biocontrol of insects. The absence of *BcAC* and *MrAC* could affect the biocontrol capacity of *B. bassiana* to *Galleria mellonella* larvae and *M. robertsii* to *Tenebrio molitor* third-instar larvae, respectively. Moreover, the environmental stress response and conidiation of the two mutants were also affected [17]. Similarly, silencing the adenylate cyclase-encoding gene *MaAC* could influence the biocontrol ability of *M. acridum* against *Locusta migratoria* adults, as well as their growth and tolerance to environmental stresses [18].

*C. rosea* 67-1 was previously isolated and exhibited excellent ability to control numerous fungal plant diseases [19]. An adenylate cyclase-encoding gene, *crac*, was previously screened from 67-1 because its expression level was dramatically upregulated during 67-1 parasitization of the sclerotia of *S. sclerotiorum* [20]. The aim of this research was to investigate the role of *crac* in *C. rosea* mycoparasitism and clarify the molecular mechanism of *C. rosea* biocontrol; in this study, the function of *crac* in *C. rosea* mycoparasitism was first investigated through gene knockout and complementation. Then, the transcriptomes of 67-1 and *crac* deletion mutant parasitizing sclerotia of *S. sclerotiorum* were sequenced and compared. Finally, differentially expressed genes after *crac* deletion were explored, and their potential roles associated with *crac* were analyzed. This study has significance for understanding the cAMP signaling pathway in *C. roses* and further improving the biocontrol ability of *C. rosea*.

## 2. Materials and Methods

### 2.1. Strains

*C. rosea* 67-1 was originally isolated from a vegetable yard in Hainan Province, China, using a sclerotia baiting method [19]. *S. sclerotiorum* Ss-H was obtained from Sclerotinia stem rot-infected soybean stems in Heilongjiang Province, China. Sclerotia of *S. sclerotiorum* were prepared and collected on carrot medium as previously described [21]. *C. rosea* 67-1 and *S. sclerotiorum* Ss-H were deposited in the Agricultural Culture Collection of China to obtain numbers ACCC 39160 and ACCC 39161, respectively.

### 2.2. Gene Cloning and Bioinformatic Analysis

A Biospin fungus genomic DNA extraction kit (Bioer Technology Co., Ltd., Hangzhou, China) was used to extract the genomic DNA of *C. rosea* 67-1. Full-length *crac* was then amplified using primer pairs AC-F and AC-R through PCR (Table 1). The PCR products were detected by electrophoresis and sequenced at Sangon Biotech Co., Ltd. (Shanghai, China) Sequence information of *crac* was submitted to GenBank to obtain accession numbers.

The ExPASy program was used to predict the molecular weight and isoelectric point of crac [22]. SignalP 6.0 and TMPRED software were used to detect the signal peptide and transmembrane regions of crac, respectively [23,24]. MEGA 6.0 software was used to conduct phylogenetic analysis of crac, with 1000 replications under the neighbor-joining method [25,26].

### 2.3. Gene Knockout

The plasmid pKH-KO, carrying a hygromycin B (*hph*) resistance gene, was used to construct a gene knockout vector. Primer pairs of CAUF/CAUR and CADF/CADR were used to amplify the upstream and downstream fragments of *crac* from the genomic DNA of *C. rosea* 67-1 (Table 1). Then, the upstream and downstream fragments, linearized pKH-KO, and USER enzyme were mixed together to construct the gene knockout vector pKH-KO-crac at 37 °C for 20 min, followed by 25 °C for 20 min, and transferred in *Escherichia coli* DH5α. Finally, the gene knockout vector pKH-KO-crac was extracted from DH5α and verified using electrophoresis and DNA sequencing.

The protoplasts of *C. rosea* 67-1 were prepared as previously described [21]. The knockout vector pKH-KO-crac was transformed into protoplasts of *C. rosea* 67-1, and the mutants were screened on potato dextrose agar (PDA) plates containing 300 μg/mL hygromycin B after 5–7 days of cultivation. The positive mutants were verified using PCR through the following strategy. First, the CDS of *crac* was amplified among the wild-type strain and mutants using primer pairs of ACF and ACR by PCR, with the wild-type strain and false-positive strains amplifying the CDS of *crac*, while the positive mutants could not amplify the CDS of *crac* through electrophoresis detection. Then, the fragments beyond the upstream and downstream of *crac* were amplified and sequenced using the primer pairs FACF and FACR, with the wild-type strain and false-positive strains amplifying a sequence length of 11,621 bp, while the positive mutants amplified 4623 bp.

Copy numbers of *hph* in knockout mutants were detected using Southern blotting to verify homologous recombination. The DNA probe was prepared with primer pairs of PACF and PACR (Table 1) and then labeled with digoxigenin (DIG) by a PCR DIG Probe Synthesis Kit (Roche) according to the manufacturer’s instructions. The genomic DNA of knockout mutants was extracted and then digested with *Sac*I (Takara) and separated using a 0.7% agarose gel. The products were transferred onto a Hybond-N+ nylon membrane (Amersham, NJ, USA) and hybridized with the labeled DNA probe. Finally, chemiluminescence was conducted using a DIG-High Prime DNA Labeling and Detection Starter Kit II (Roche, Penzberg, Germany). Finally, mutants with single band through Southern blot were regarded as positive.

### 2.4. Gene Complementation

The plasmid pKN, containing a G418 resistance gene, was used to construct the gene complementation vector. Primer pairs of HBACF and HBACR were used to amplify the complementation fragments of *crac* using PCR (Table 1). The gene complementation vector was constructed through linkage of *crac* complementation fragments and the linearized pKN plasmid, and then the complementation vector pKN-crac was verified by electrophoresis and vector sequencing.

The complementation vector was transformed into the protoplasts of knockout mutants, and then complementation transformants were screened on potato dextrose agar (PDA) plates containing 300 μg/mL G418. The positive transformants were verified using PCR with primer pairs AC-F and AC-R (Table 1), with the positive transformants amplifying the CDS of *crac*.

### 2.5. Morphological Characteristics and Stress Tolerance

The *C. rosea* wild-type strain, knockout mutants, and complementation transformants were inoculated on PDA plates at 26 °C for 10 days. Then, differences in colony morphology and growth rate among the three types of *C. rosea* strains were examined at the same amount of time growing.

NaCl and H_2_O_2_ were used to test the tolerance alteration when *crac* was absent and complementary in *C. rosea* 67-1 under environmental stresses. The *C. rosea* wild-type strain, knockout mutants, and complementation transformants were inoculated on PDA plates containing 1 M NaCl and 20 mM H_2_O_2_. The colony diameters of the three types of *C. rosea* strains were measured at 26 °C for 10 days. Each treatment was conducted in triplicate.

### 2.6. Mycoparasitic Ability on Sclerotia of S. sclerotiorum

The spore concentrations of the *C. rosea* wild-type strain, knockout mutants, and complementation transformants were adjusted to 1 × 10^7^ spores/mL. Sclerotia of *S. sclerotiorum* were surface-sterilized with 75% ethyl alcohol and 1% NaClO and then immersed into the spore suspensions of *C. rosea* strains for 10 min. Then, sclerotia from different types of *C. rosea* strains were placed on wet filter paper in a Petri dish at 26 °C for 7 days. The infection severity of *C. rosea* strains parasitizing sclerotia was investigated under a 4-grade scale: 0 = no *C. rosea* mycelium were found on the sclerotia; 1 = few *C. rosea* mycelia were found on the sclerotia; 2 = sclerotia were covered with *C. rosea* mycelia but not soft; 3 = sclerotia were covered with *C. rosea* mycelia and soft [4]. All treatments were repeated three times.

### 2.7. Biocontrol of Soybean Sclerotinia Stem Rot

Soybeans were planted in pots and grown to nine compound leaves for the biocontrol assay. Spore suspensions with equal concentrations (5 × 10^6^ spores/mL) of the *C. rosea* wild-type strain, knockout mutants, and complementation transformants were sprayed onto soybean leaves. Then, an equivalent amount of *S. sclerotiorum* suspension was inoculated after 2 h. The negative control in the experiment was sterile distilled water sprays and the positive control was application of the fungicide carbendazim according to supplier’s instructions. The disease index of soybean Sclerotinia stem rot was investigated using a 9-grade scoring system after 7 days based on the lesion areas on soybean leaves: 0, no symptoms; 1, <5%; 3, 5–10%; 5, 11–25%; 7, 26–50%; and 9, > 50%. The disease index was calculated as follows: disease index = [Σ (number of diseased leaves × disease grade)/(total number of leaves × the highest disease grade)] × 100 [21]. The control efficacies of *C. rosea* strains were also assessed as follows. Control efficacy (%) = [(Disease index of negative control-disease index of experimental group)/disease index of negative group] × 100. All experiments were performed with three replications.

### 2.8. Preparation of Mycelial Samples of C. rosea Strains Mycoparasitizing Sclerotia

The concentration of spore suspensions of the *C. rosea* wild-type strain and knockout mutant was adjusted to 1 × 10^7^ spores/mL. A total of 100 μL of spore suspensions of the wild-type strain and knockout mutant were spread on PDA plates using a sterilized glass scraper and cultivated at 26 °C for 48 h. Surface-sterilized sclerotia with uniform size were placed onto and covered the surface of the wild-type strain and knockout mutant plates and cultivated at 26 °C. The sclerotia were removed at 8, 24, and 48 h. Then, mycelia of *C. rosea* wild-type strains and *crac* knockout mutants were collected at 8, 24, and 48 h using a sterilized spatula used for RNA extraction. Mycelia of *C. rosea* wild-type strains parasitizing sclerotia were used as control. Set mycelia of *C. rosea crac* knockout mutants parasitizing sclerotia were used as treatment. Each treatment was conducted three times.

### 2.9. cDNA Library Construction and Sequencing

Total RNA of each mycelia sample was extracted, and the quality of RNAs was detected using an Agilent 2100 bioanalyzer. mRNA from each sample was enriched with oligo (dT) magnetic beads and then broken into small pieces using NEB fragmentation buffer. cDNA fragments with a size of 250–300 bp were selected using AMPure XP beads after cDNA synthesis, purification, end repair, addition of adenine to the 3′ end, and adapter connection. Finally, suitable cDNA fragments were amplified to construct cDNA libraries. The cDNA libraries were monitored and sequenced using the Illumina NovaSeq 6000 platform (Illumina Inc., San Diego, CA, USA) at Novogene Corporation Inc.

Raw transcriptome sequencing data were filtered to remove adaptor sequences, reads that contained N (unknown bases), and low-quality reads to obtain clean data. HISAT software (v2.0.5) was used to map the clean data onto the genome of *C. rosea* 67-1. The reads were submitted to the NCBI Sequence Read Archive (SRA) database to obtain accession numbers.

### 2.10. Differentially Expressed Gene Analysis

The FPKM (expected number of fragments per kilobase of transcript sequence per million base pairs sequenced) method was used to calculate the expression level of genes [27,28]. The differentially expressed genes (DEGs) after *crac* deletion in *C. rosea* strains mycoparasizing sclerotia were screened using DESeq2 software (1.20.0) according to negative binomial distribution [29]. A padj value lower than 0.05 and a fold change higher or lower than 2 were considered significantly differentially expressed. Finally, Gene Ontology (GO) and Kyoto Encyclopedia of Genes and Genomes (KEGG) pathway enrichment analyses were conducted for the DEGs.

### 2.11. Quantitative Real-Time PCR Validation

To validate the reliability of transcriptomic sequencing, the expression level of 8 genes, containing 4 upregulated expressed and 4 downregulated expressed genes, were randomly selected and quantified through quantitative real-time PCR. Primer pairs of the 8 genes were designed using Primer Premier 6.0 software (Table S1).

The first strand cDNA of mRNA samples was synthesized using a cDNA synthesis kit (TAKARA) according to the producer’s instruction. Quantitative real-time PCR was conducted using CFX96 Real-Time PCR Detection System, with the program at 95 °C 30 s; 40 cycles of 95 °C 5 s, and 55 °C 30 s. The 2^−∆∆Ct^ method was used to calculate the expression levels of genes, with elongation factor used as internal reference gene. Each experiment was performed in three replications.

### 2.12. Statistical Analysis

SAS 9.1.3 software (SAS Institute Inc., Cary, NC, USA) was used to analyze the differences in biological characteristics, tolerance to different stresses, mycoparasitic ability to sclerotia, and biocontrol capacity of soybean Sclerotinia stems among the *C. rosea* wild-type strain, knockout mutants, and complementation transformants. Duncan’s multiple range test was used to compare the means of each treatment. An amount of *p* < 0.05 was regarded as significant.

## 3. Results

### 3.1. Characterization of Crac

**Figure 1:**
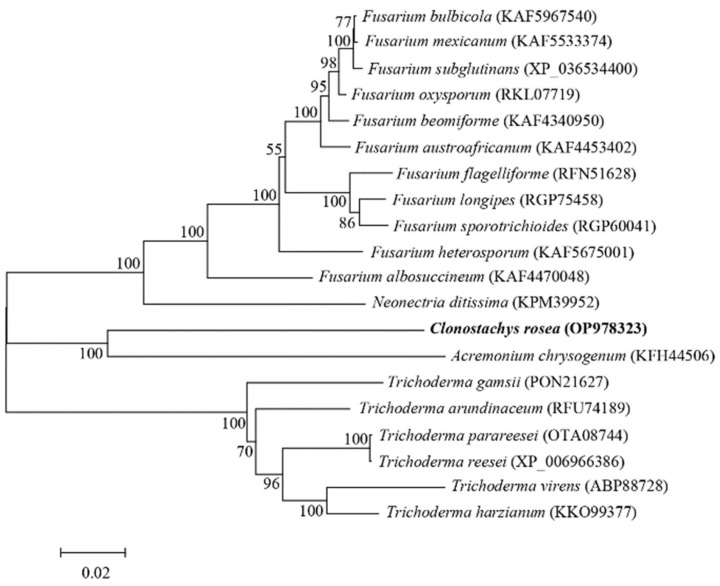
Phylogenetic analysis of crac from *Clonostachys rosea* using the neighbor-joining method. Numbers at the nodes indicate the bootstrap values of 1000 bootstraps. Bars (=0.02) represent sequence divergence.

The complete sequence of *crac* comprises 8477 bp and contains 1220 bp of intronic DNA. The predicted crac protein consisted of 2418 amino acids, with an isoelectric point and predicted molecular weight of 5.81 and 265.4 kDa, respectively. No signal peptide or transmembrane regions were detected within the crac protein. The phylogenetic analysis found that the crac protein was clustered with other closely related adenylate cyclases, indicating that crac from *C. rosea* belongs to the adenylate cyclase group (Figure 1). The complete sequence of *crac* was submitted to GenBank and obtained accession number OP978323.

### 3.2. Screening and Verification of Crac Knockout Mutants and Complementation Transformants

Upstream and downstream fragments of *crac* were amplified through PCR and linked to the linearized pKH-KO plasmid. Complementation fragments of *crac* were also amplified through PCR and linked to the linearized pKN plasmid. The knockout and complementation vectors were successfully constructed through electrophoresis and plasmid sequencing verification.

**Figure 2:**
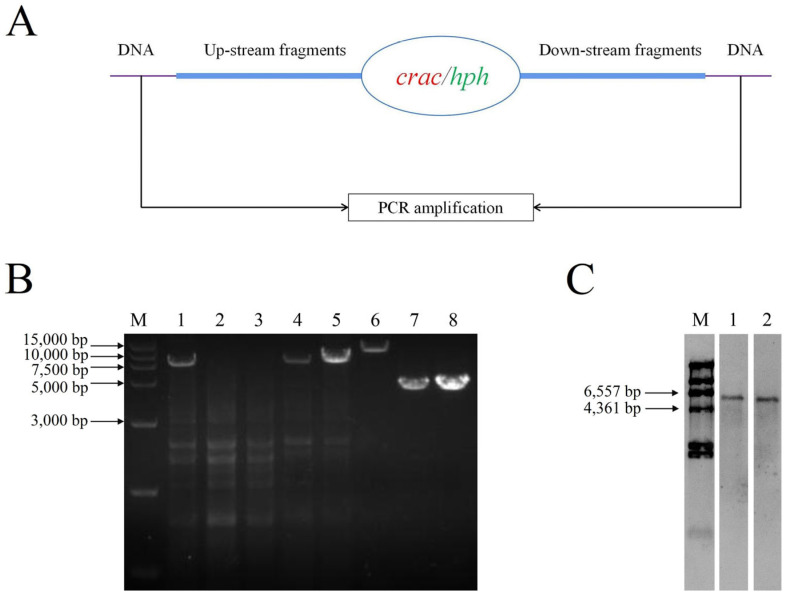
Verification of *crac* knockout mutants and complementation transformants. (**A**) Verification strategy of *crac* knockout mutants. (**B**) PCR verification. M: Marker; 1–5: cds of *crac* in the wild strain, *Δcrac*-6, *Δcrac*-13, *Δcrac*-6+, and *Δcrac*-13+; 6–8: fragment beyond the upstream and downstream region in the wild strain, *Δcrac*-6, and *Δcrac*-13. (**C**) Number of *hph* copies detected by Southern blotting. M: Marker; 1: *Δcrac*-6; 2: *Δcrac*-13.

The *crac* knockout vector was transformed into the *C. rosea* wild-type strain, and two positive mutants (named ∆*crac-6* and ∆*crac-13*) were obtained and verified through PCR amplification and sequencing (Figure 2). The results of Southern blotting demonstrated that only one copy of the hph fragment was detected in both the ∆*crac-6* and ∆*crac-13* mutants, which indicated that *crac* was deleted through homologous recombination in *C. rosea* 67-1.

The *crac* complementation vector was transformed into the *C. rosea* ∆*crac-6* and ∆*crac-13* mutants, and then complementation transformants named ∆*crac-6*+ and ∆*crac-13*+ were obtained and verified through PCR amplification and sequencing (Figure 2).

### 3.3. Morphological Characteristics and Stress Tolerance

**Figure 3:**
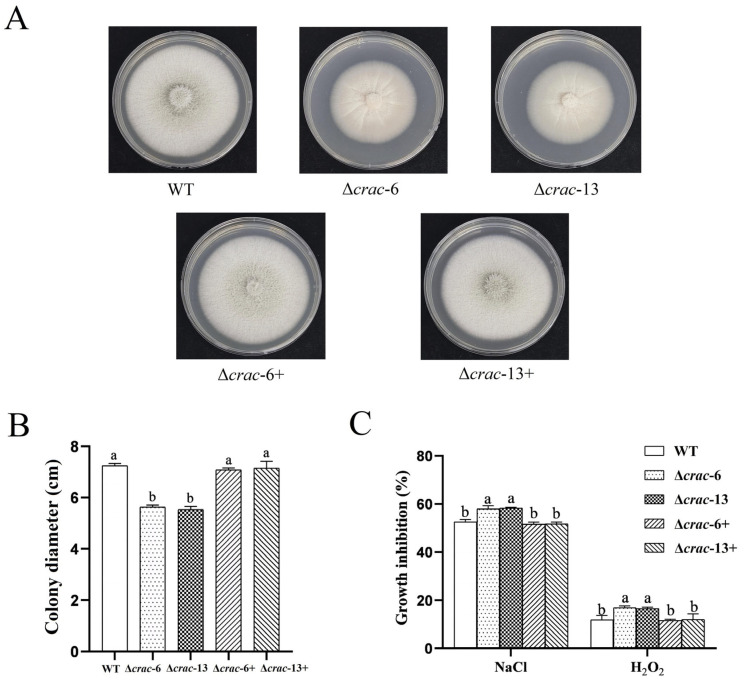
Morphological characteristics among the wild strain, *crac* knockout mutants, and complementation transformants. (**A**) Colony morphology. (**B**) Growth diameter. (**C**) Tolerance to stress of NaCl and H_2_O_2_. Different letters in Figure (**B**) represent significant difference (*p* < 0.05) in mycelial diameter (cm). Different letters in Figure (**C**) represent significant difference (*p* < 0.05) in growth inhibition (%) under different environmental stress. Error bars represent the standard deviation (SD) of three experiments.

The colony morphology of *C. rosea* was altered in the ∆*crac-6* and ∆*crac-13* mutants compared with the wild-type strain. The mycelium of *C. rosea* on the colony became lesser after *crac* deletion. The colony morphology was recovered after crac complementation in the two mutants (Figure 3A). Colony diameters were significantly decreased after *crac* deletion in both *C. rosea* knockout mutants. After gene complementation, the colony diameters of ∆*crac-6*+ and ∆*crac-13*+ showed no significant difference from the wild-type strain (Figure 3B).

In the stress tolerance assay, the tolerance of *C. rosea* to NaCl and H_2_O_2_ was dramatically reduced after *crac* deletion. The inhibition rate of NaCl and H_2_O_2_ in both ∆*crac-6* and ∆*crac-13* was significantly higher than that in the wild-type strain and complementation transformants (Figure 3C), indicating that *crac* is associated with tolerance to stress.

### 3.4. Mycoparasitic Ability on Sclerotia of S. sclerotiorum

**Figure 4:**
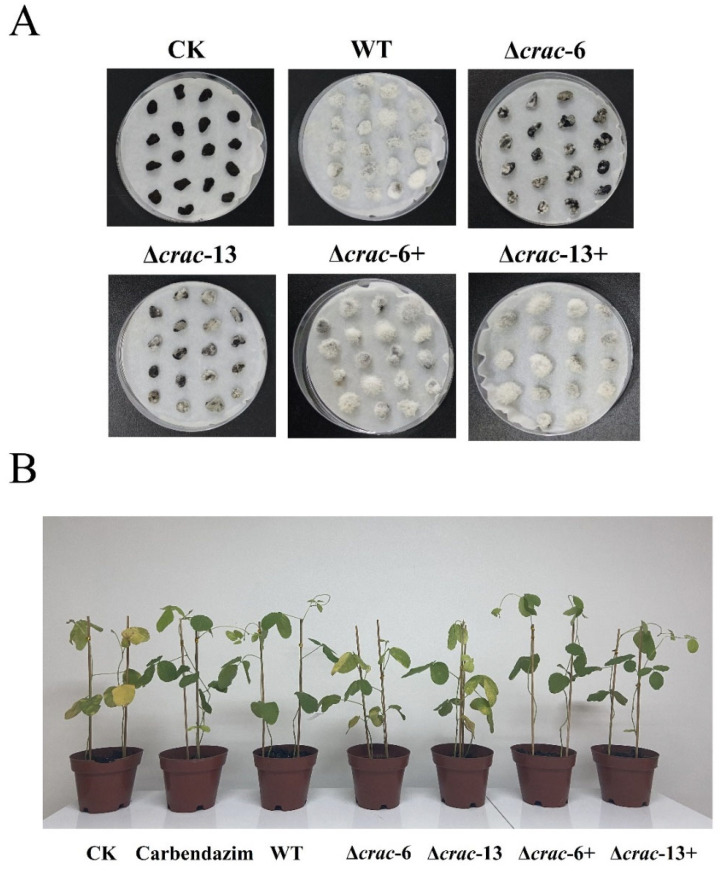
Mycoparasitic ability and biocontrol capacity among the wild strain, *crac* knockout mutants, and complementation transformants. (**A**) Mycoparasitic ability on sclerotia of *S. sclerotiorum*. (**B**) Biocontrol capacity on soybean Sclerotinia stem rot. CK: treatment with distilled water; WT represents wild-type strain.

The infection severity of sclerotia was reduced in both ∆*crac-6* and ∆*crac-13* mutants compared with the wild strain after 7 days of cultivation. Sclerotia that were parasitized by the *C. rosea* wild strain were rotten and reached grade 3. However, sclerotia that were parasitized by ∆*crac-6* and ∆*crac-13* were still firm and reached grade 2. After *crac* complementation, the infection severity of sclerotia by ∆*crac-6*+ and ∆*crac-13*+ was restored to grade 3, which suggested that *crac* was crucial in *C. rosea* mycoparasitism (Figure 4).

### 3.5. Biocontrol of Soybean Sclerotinia Stem Rot

Serious soybean Sclerotinia stem rot diseases occurred after 7 days in soybean seedlings in the CK treatment (sprayed with sterile distilled water) after inoculation with *S. sclerotiorum*. The wild strain exhibited excellent biocontrol efficacy against soybean Sclerotinia stem rot (74.1%), which is similar to the biocontrol effects of carbendazim (72.6%). However, the control efficacy was significantly reduced in the treatment of ∆*crac-6* and ∆*crac-13* (34.2% and 31.6%, respectively). The control efficacy in the complementation transformants ∆*crac-6*+ and ∆*crac-13*+ recovered and showed no remarkable difference with the treatment of the wild strain (71.1% and 69.1%, respectively), which demonstrated that *crac* plays important roles in *C. rosea* biocontrol (Figure 4, Table 2). 

### 3.6. Transcriptome Sequencing

After transcriptome sequencing and data filtration, the average number of clean reads in the samples was 43,250,676. All samples were mapped onto the genome of *C. rosea* 67-1, and the map rate was above 93.46% for each sample. All sequence data were uploaded to SRA with the accession numbers SRR22518306, SRR22525956, SRR22540850, SRR22541076, SRR22558352, SRR22572162, SRR22581774, SRR22585856, SRR22671486, SRR22673279, SRR22684623, SRR22702311, SRR22704875, SRR22727039, SRR22730640, SRR22799385, SRR22805652, and SRR22512441.

### 3.7. DEG Analysis

Through transcriptomic analysis, the *crac* gene in the wild strain could be detected at three sampling times, but the *crac* gene could not be detected in both of the *crac* deletion mutants at three sampling time points. After *crac* deletion, 1623 and 1371 genes were significantly upregulated and downregulated in *C. rosea* at 8 h parasitizing sclerotia based on transcriptomic analysis. At 24 h mycoparasitism, the upregulated and downregulated DEGs were 281 and 355, respectively. At 48 h mycoparasitism, the upregulated and downregulated DEGs were 359 and 231, respectively. Among these DEGs, a number of genes with unknown functions dominated at 8, 24, and 48 h. Genes encoding different types of transporters, including sugar transporters, ABC transporters, transmembrane amino acid transporters, oligopeptide transporters, siderophore iron transporters, major facilitator superfamily multidrug transporters, monocarboxylate transporters, vitamin B6 transporters, and riboflavin transporters, were significantly downregulated during the process of *C. rosea* parasitizing sclerotia after *crac* disruption. Similarly, genes encoding cell wall-degrading enzymes, including chitinase, endoglucanase and protease, cytochrome P450 monooxygenase, and nonribosomal peptide synthetase, were also dramatically downregulated after *crac* deletion.

**Figure 5:**
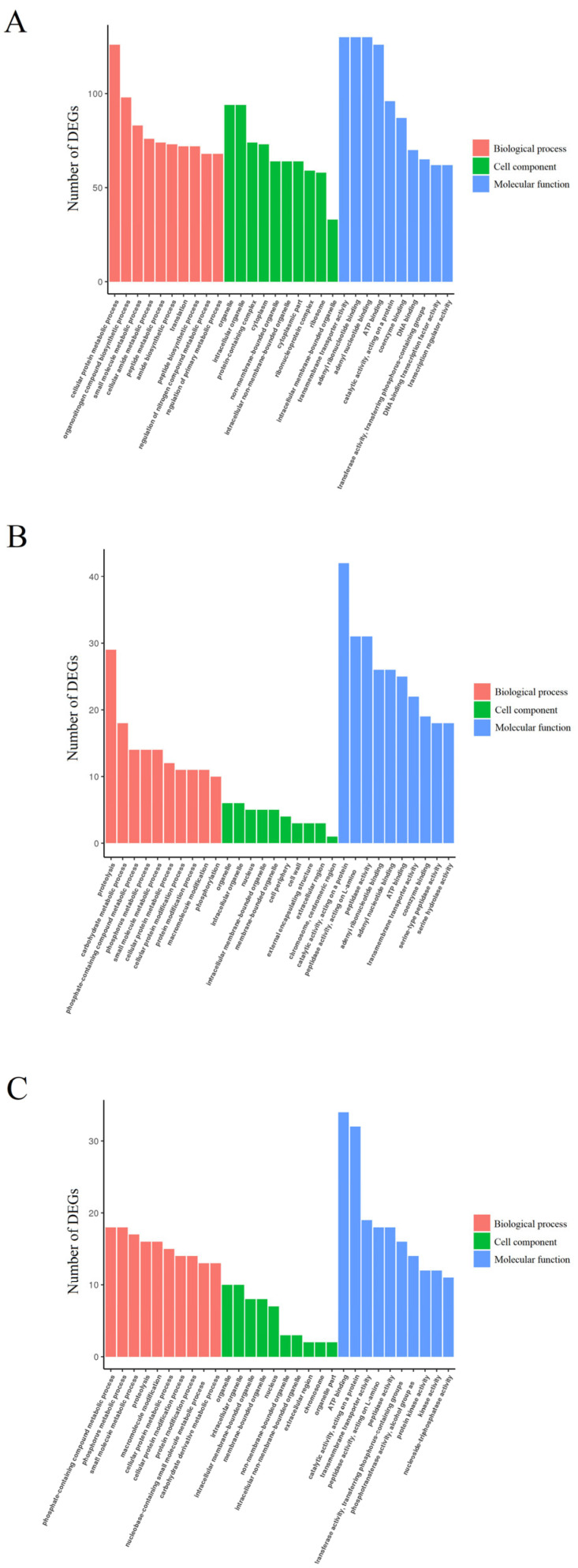
Gene ontology (GO) categories of the DEGs. (**A**) 8 h mycoparasitism. (**B**) 24 h mycoparasitism. (**C**) 48 h mycoparasitism. The *X*-axis represents different GO teams of DEGs belonging to three categories. The *Y*-axis means number of DEGs.

According to GO enrichment analysis, the functions of DEGs were divided into three categories: biological process, cellular component, and molecular function (Figure 5). At 8 h mycoparasitism, the cellular protein metabolic process term belongs to the biological process category; organelle and intracellular organelle terms belong to the cellular component category; and transmembrane transporter activity, adenyl ribonucleotide binding, and adenyl nucleotide binding terms belong to the molecular function category. The dominant terms in the cellular component category were not altered at 24 and 48 h. However, the dominant terms in the biological process and molecular function categories were quite different at 8, 24, and 48 h. At 24 h, the dominant terms in the biological process and molecular function categories were proteolysis and catalytic activity, acting on a protein, respectively. At 48 h, the dominant terms in the biological process and molecular function categories were phosphate metabolic process and ATP binding, respectively.

**Figure 6:**
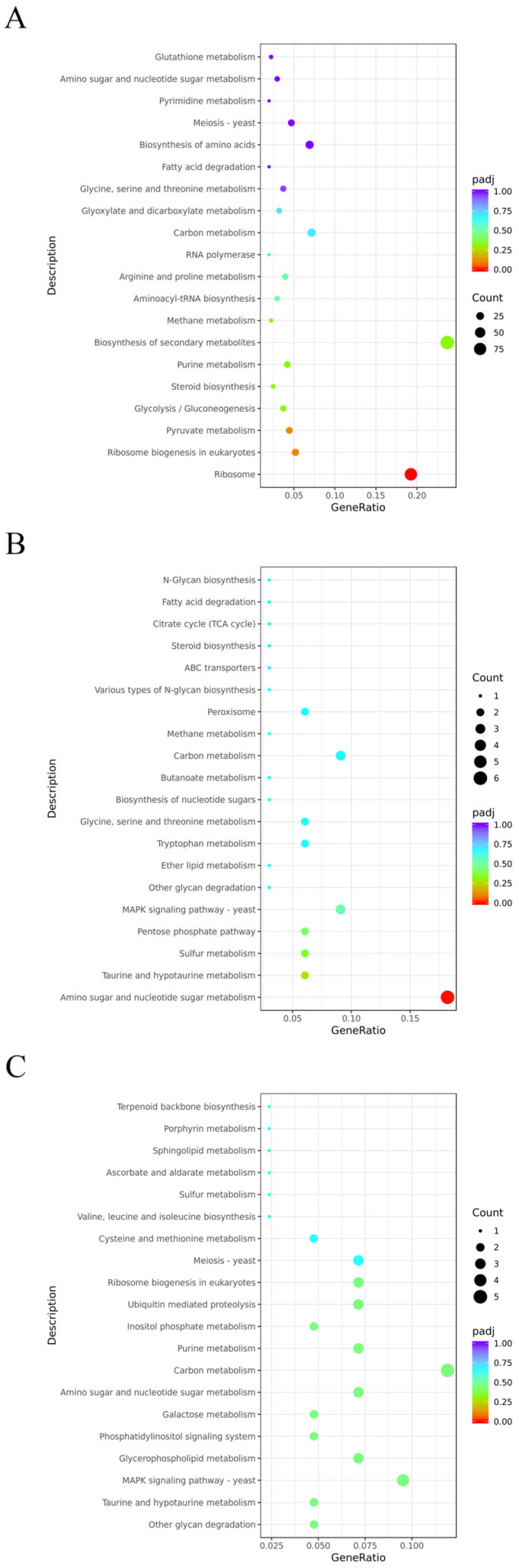
KEGG analysis of the DEGs. (**A**) 8 h mycoparasitism. (**B**) 24 h mycoparasitism. (**C**) 48 h mycoparasitism. The *X*-axis represents the ratio of DEGs annotated onto KEGG pathways/all DEGs. *Y*-axis means different types of KEGG pathways. The areas of circle represent number of DEGs, and the color of circle from red to purple represents significance value ranging from lower to higher.

KEGG analysis was found to be helpful for understanding the pathways of DEGs involved during *C. rosea* mycoparasitism (Figure 6). At 8 h, 96 DEGs were involved in secondary metabolite biosynthesis pathways. At 24 h, the most dominant pathway was amino sugar and nucleotide sugar metabolism. At 48 h, the most dominant pathway was carbon metabolism. According to KEGG analysis, some DEGs were involved in penicillin and cephalosporin biosynthesis or chitin degradation, which is the main component of the pathogen cell wall. These findings indicated that *crac* might affect the capacity of antibiotic production or cell wall degradation by regulating the expression levels of these DEGs, therefore influencing the mycoparasitic ability of *C. rosea*.

### 3.8. Quantitative Real-Time PCR Validation

**Figure 7:**
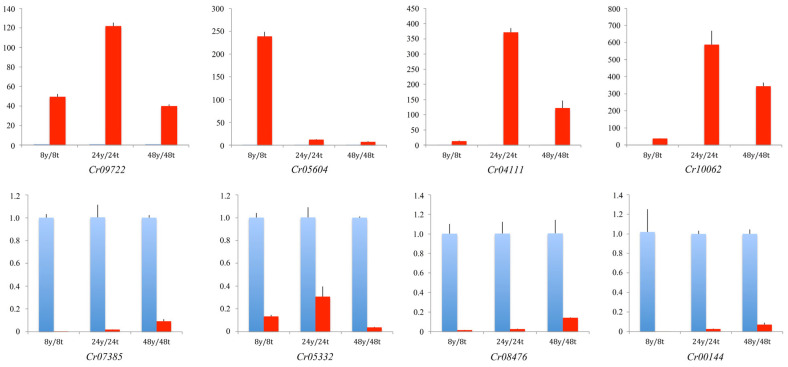
Validation of the DEGs using quantitative real-time PCR. The *X*-axis represents DEGs, and the *Y*-axis represents relative expression level of DEGs. Blue column represents the wild strain, and red column represents the crac knockout mutant. The top and bottom panel means up and downregulated DEGs, respectively. Error bars indicated the standard deviation (SD) of three replications.

Among these DEGs, eight genes, including four upregulated and four downregulated genes, were randomly selected to conduct quantitative real-time PCR. The results showed that the expression level of the eight genes was consistent with transcriptomic analysis during all the time points, which indicated that the expression level of DEGs from transcriptomic sequencing was reliable for further analysis (Figure 7).

## 4. Discussion

The signal transduction pathways play crucial roles in mycoparasitism. Molecular signals are cascade-amplified through signal transduction pathways and then activate downstream effectors, accomplishing the mycoparasitism behavior [30]. Therefore, exploring signal transduction pathways is important for further understanding the mycoparasitism mechanism of mycoparasites. MAPK and cAMP are the major signal transduction pathways in fungi. Our previous study analyzed the role of some important genes in the MAPK signal transduction pathway during mycoparasitism [2]. As another important signal transduction pathway, the cAMP pathway has seldom been studied in biocontrol. In our previous work, the adenylate cyclase-encoding gene *crac*, as an important component in the cAMP pathway, was significantly upregulated during *C. rosea* mycoparasitism [20]. Therefore, to explore the role of *crac* in *C. rosea* mycoparasitism, gene knockout and complementation were used to analyze the function of *crac*. The results obtained that the colony morphology and tolerance to environmental stress were influenced after the deletion of *crac*, as well as the parasitic activity of *C. rosea* on sclerotia of *S. sclerotiorum* and the biocontrol capacity of *C. rosea* against soybean Sclerotinia stem rot. In order to clarify whether the mycoparasitic ability decline was due to *crac* deletion, or associated with general fitness defects, and further study the molecular mechanism by which *crac* regulates *C. rosea* mycoparasitism, transcriptomes of the *C. rosea* wild-type strain and knockout mutant parasitizing sclerotia were sequenced, and the DEGs after *crac* deletion were analyzed. Numerous genes were significantly altered in expression after *crac* deletion; among these DEGs, mycoparasitic-related genes such as cell wall degrading enzymes were significantly lower, which indicated that *crac* was involved in *C. rosea* mycoparasitism. This study provides new insights into the mycoparasitism mechanism of *C. rosea*.

As a crucial component in the cAMP signal transduction pathway, adenylate cyclase plays important roles in regulating morphological characteristics, vegetative growth, conidiation, sexual development, mating, and resistance to environmental stress in fungi, as well as metabolite production. Moreover, adenylate cyclase has been associated with pathogenicity in fungal pathogens. Disruption of adenylate cyclase genes in *S. sclerotiorum*, *Ustilaginoidea virens*, *Botrytis cinerea*, *Penicillium digitatum,* and *Magnaporthe grisea* could attenuate their pathogenicity to tomato leaflets, rice seeds and panicles, *Phaseolus vulgaris* leaves, citrus fruit, and rice leaves, respectively [31,32,33,34,35]. However, only a few studies of adenylate cyclase involved in biocontrol have been reported. Deletion and silencing of adenylate cyclase genes in *T. virens* and *M. acridum* reduced their biocontrol ability against pathogens [16,17]. To date, no study of adenylate cyclase in *C. rosea* mycoparasitism has been reported. Therefore, studying the role of adenylate cyclase in *C. rosea* mycoparasitism has significance for deeply exploring the biocontrol mechanism of *C. rosea*.

Analysis of the DEGs aids in understanding the mechanism by which *crac* regulates *C. rosea* mycoparasitism. In this study, 1623 and 1371 genes were significantly upregulated and downregulated in *C. rosea* parasitizing sclerotia at 8 h after crac deletion based on transcriptomic analysis. However, at 24 and 48 h, the numbers of DEGs were markedly reduced compared with those at 8 h. At 24 h, there were 281 and 355 upregulated and downregulated DEGs, respectively. At 48 h mycoparasitism, the upregulated and downregulated DEGs were 359 and 231, respectively. This may be associated with the property of *crac*, which belongs to a component of the cAMP signal pathway. Signal transduction commonly occurs at the initial stage of mycoparasitism. As a signal transduction-related gene, deletion of *crac* would likely lead to more DEG alterations at the initial stage (8 h) of *C. rosea* mycoparasitism compared with 24 and 48 h.

Among the DEGs, several different types of genes have received considerable attention, including transporter and cell wall-degrading enzymes, which have been reported to be involved in biocontrol. Deletion of the ABC transporter-encoding gene *abcG29* in *C. rosea* reduced the ability to protect *Arabidopsis thaliana* leaves infected by *B. cinerea* [9]. Cell wall-degrading enzymes normally refer to chitinase, glucanase, and protease, which can degrade the cell wall of fungal pathogens. Overexpression of the endochitinase-encoding gene Chi67-1 in *C. rosea* was shown to enhance the mycoparasitic ability to sclerotia and biocontrol capacity to soybean Sclerotinia stem rot [21]. Deletion or overexpression of the beta-1,6-glucanase Tvbgn3 in *T. virens* significantly reduced and increased the biocontrol ability, respectively [36]. Similarly, disruption of the subtilisin-like protease prC attenuated the nematode infection ability of *C. rosea* [37]. Moreover, different types of monooxygenase and nonribosomal peptide synthetase also participate in biocontrol. Disruption of the monooxygenase-encoding gene in *T. hamatum* caused a reduced ability to inhibit *S. sclerotiorum* growth and sclerotial production [38]. Deletion of the nonribosomal peptide synthetase-encoding gene nps1 reduced the biocontrol ability of *C. rosea* against wheat foot rot disease caused by *Fusarium graminearum* [6]. In our study, these DEGs were significantly downregulated after crac deletion, which indicated that *crac* might be involved in *C. rosea* mycoparasitism by regulating the expression of these DEGs.

Moreover, several DEGs encoding transcription factors were significantly downregulated during *C. rosea* after *crac* deletion. Transcription factors are downstream components of signal transduction pathways. When signals are cascade-amplified through signal transduction pathways, transcription factors become active and combine with related cis-acting elements to regulate the expression of target genes and response to related biological behavior [39]. Transcription factor-encoding genes have been reported to be associated with biocontrol. Disruption of the transcription factor gene crtf in *C. rosea* could reduce the mycoparasitic ability of sclerotia and biocontrol capacity to soybean Sclerotinia white mold [4]. Similarly, deletion of the NDT80-like transcription factor gene CmNdt80a could significantly diminish the mycoparasitic ability of *Coniothyrium minitans* on sclerotia of *S. sclerotiorum* [40]. In this study, some downregulated transcription factor genes were involved in the biosynthesis of secondary metabolites through KEGG analysis, which indicated that *crac* might affect secondary metabolite production by regulating the expression of transcription factor genes, thereby influencing the mycoparasitic ability of *C. rosea*. Further study could explore the function of these transcription factor genes in *C. rosea* biocontrol and seek their cis-acting elements associated with biocontrol.

## 5. Conclusions

Deletion of *crac* influenced the colony morphology and growth rate of *C. rosea*, as well as the tolerance of NaCl and H_2_O_2_ stress. The mycoparasitic ability of *C. rosea* on sclerotia of *S. sclerotiorum* and the biocontrol capacity of *C. rosea* on soybean Sclerotinia stem rot were both attenuated in *crac* knockout mutants. All characteristics were recovered after gene complementation, which indicated that crac was involved in *C. rosea* biocontrol. Moreover, the deletion of *crac* caused numerous biocontrol-related genes to be differentially expressed, including genes encoding cell wall-degrading enzymes, transporters, monooxygenase, etc. This study provides a basis for further deep exploration of the molecular mechanism of *C. rosea* mycoparasitism.

## Figures and Tables

**Table 1:** Primers in this study.

Primers	Sequence (5′-3′)
CAUF	GGTCTTAAUCGCCGTGGGATGGATTGGACGAGGA
CAUR	GGCATTAAUTGTTCAACCTACGGCCGTAGCTGGC
CADF	GGACTTAAUGAAGTAGTAGTGCCAGGGCGACACG
CADR	GGGTTTAAUCGACCGACTGCGAGAGATTGAAACT
AC-F	ATGACCAAAAATGAGGCCGTGAG
AC-R	TCATCCGCCCTCCCTCGCCTCAT
FACF	ACCTCCATAAAGGCGTAGGACAGAC
FACR	TGCTCAGCCTCCCTACAGACAACCT
PACF	CGTTATGTTTATCGGCACT
PACR	TTGGCGACCTCGTATTGG
HBACF	TGGATCCCCCGGGCTGCAGGAATTCTTGGCGGGCTATTGACTTATGATAA
HBACR	CGAGGTCGACGGTATCGATAAGCTTCGACCGACTGCGAGAGATTGAAACT

**Table 2:** Control efficacy of *Clonostachys rosea* strains against soybean Sclerotinia stem rot.

Strains	Disease Index	Control Efficacy (%)
CK	75.6	-
WT	19.6	74.1 ^a^
Carbendazim	20.7	72.6 ^a^
∆*crac-6*	49.7	34.2 ^b^
∆*crac-13*	45.9	31.6 ^b^
∆*crac-6* +	22.7	71.1 ^a^
∆*crac-13* +	23.3	69.1 ^a^

CK: treatment with sterilized water; WT: wild-type strain. Note: Different letters represent significant difference, *p* < 0.05.

## Data Availability

All sequence data were uploaded to SRA with the accession numbers SRR22518306, SRR22525956, SRR22540850, SRR22541076, SRR22558352, SRR22572162, SRR22581774, SRR22585856, SRR22671486, SRR22673279, SRR22684623, SRR22702311, SRR22704875, SRR22727039, SRR22730640, SRR22799385, SRR22805652, and SRR22512441.

## Supplementary Materials

The following supporting information can be downloaded at: https://www.mdpi.com/article/10.3390/jof9080861/s1. Table S1. Primers used for quantitative real-time PCR.
