# Supplementary material for: The Adenylate Cyclase-Encoding Gene crac Is Involved in Clonostachys rosea Mycoparasitism
Source: J Fungi (Basel). 2023 Aug 18;9(8):861. doi: 10.3390/jof9080861 (PMC10455997; doi:10.3390/jof9080861)
Supplement: Supplementary file 1 [file jof-09-00861-s001.zip › jof-2530336-supplementary.pdf]

Table S1. Primers used for quantitative real-time PCR .

Primers	Sequence (5'-3')
EF1F	TCGATGTCGCTCCTGACT
EF1R	AGCGTGACCGTTTATTTGA
Cr09722F	CTTGACCAGGTTCTACGG
Cr09722R	GCAGACCAGTTACCCTTCTAT
Cr04111F	TGGGAGAAGAAAGAATGGG
Cr04111R	GGAGTAGTGTCGGACTGAGAA
Cr05604F	GGGCGGAGAAGGCTCGTAA
Cr05604R	GCATCTGGGTCGTCTGAAC
Cr10062F	GCACGCAAGTGATGAAGG
Cr10062R	TTATGCGGATGATGAGGC
Cr07385F	ATTGGCTTGCTATTGAGAT
Cr07385R	GTAATGGTTCCCTTGGTG
Cr08476F	GTCTGAGGCAGGCGAGTT
Cr08476R	CCAAATATGCACGATGTCC
Cr05332F	TCTCCCTGTCGTTGAAGT
Cr05332R	GCTGGTCGCTGTTTAGAT
Cr00144F	TTGATTGGAAACGGATTGA
Cr00144R	GTCGTAGCAGCCTTGGAT
